# Vector-borne pathogens in dogs of different regions of Iran and Pakistan

**DOI:** 10.1007/s00436-020-06992-x

**Published:** 2021-01-28

**Authors:** Roberta Iatta, Alireza Sazmand, Viet-Linh Nguyen, Farzad Nemati, Muhammad Mazhar Ayaz, Zahra Bahiraei, Salman Zafari, Anna Giannico, Grazia Greco, Filipe Dantas-Torres, Domenico Otranto

**Affiliations:** 1 Department of Veterinary Medicine, University of Bari, Bari, Italy; 2 Department of Pathobiology, Faculty of Veterinary Science, Bu-Ali Sina University, Hamedan, Iran; 3 Zoonotic Diseases Research Center, School of Public Health, Shahid Sadoughi University of Medical Sciences, Yazd, Iran; 4 Department of Parasitology, Faculty of Veterinary Sciences, Cholistan University of Veterinary and Animal Sciences, Bahawalpur, Pakistan; 5 Istituto Zooprofilattico della Puglia e della Basilicata, Putignano (BA), Italy; 6 Department of Immunology, Aggeu Magalhães Institute, Oswaldo Cruz Foundation (Fiocruz), Recife, Brazil

**Keywords:** *Anaplasma platys*, Canine vector-borne pathogens, *Ehrlichia canis*, *Rickettsia* spp., *Hepatozoon canis*, *Leishmania infantum*, Iran, Pakistan

## Abstract

Canine vector-borne diseases (CVBDs) are highly prevalent in tropical and subtropical countries, mainly due to favorable climate conditions and reduced adoption of preventive measures. This study aimed to provide a comprehensive overview on the prevalence of CVBDs in Iran and Pakistan where limited data are available. Blood samples were collected from 403 dogs from six provinces in Iran and Pakistan to assess the presence of pathogen DNA (i.e., *Anaplasma* spp., *Coxiella burnetii*, *Ehrlichia* spp., *Rickettsia* spp., *Babesia* spp*.*, *Hepatozoon* spp., filarioids, and *Leishmania* spp.). Sera were also screened by an immunofluorescence antibody test for the detection of antibodies against *Leishmania infantum*. In total, 46.9% of dogs scored positive to *Hepatozoon canis* being the most frequently detected (41.4%), followed by *Anaplasma platys* (6.4%), *Ehrlichia canis* (3.4%), *Rickettsia* spp. (2.2%), *Babesia vogeli* (1.0%), and *L. infantum* (0.3%). A seroprevalence of 9.6% to anti-*L. infantum* IgG was also recorded. Data reported herein demonstrate that dogs from Iran and Pakistan are at a high risk of CVBDs, particularly of canine hepatozoonosis. Effective control strategies are advocated for minimizing the risk of infection in animals and humans, also in consideration of the zoonotic potential of some pathogens detected.

## Introduction

Vector-borne diseases (VBDs) are of growing concern to people across the world and their increasing incidence has been attributed to several factors, such as climate change and animal movements (Ogden and Lindsay 2016). Their distribution depends on a complex combination of biotic and abiotic factors, making their control extremely difficult (Dantas-Torres et al. 2012; Otranto et al. 2017). The impact of VBDs is heavier on tropical and subtropical countries, where the climate is more suitable for various arthropod vectors and where people and animals have limited access to healthcare services, including diagnosis and treatment of these diseases (Otranto et al. 2017; Colella et al. 2019).

Dogs serve as blood source to arthropod vectors and are suitable reservoirs of many vector-borne pathogens (VBPs; Otranto 2018). For example, dogs are well recognized as the primary reservoirs of *Leishmania infantum*, the causative agent of zoonotic visceral leishmaniasis and the target of integrated control strategies (Travi et al. 2018; Dantas-Torres et al. 2019). Also, dogs are primary reservoirs of some mosquitoes-transmitted filarial helminths (e.g., *Dirofilaria immitis* and *Dirofilaria repens*), which may pose a risk to humans in areas where dogs are highly infected, including municipal shelters (Panarese et al. 2020). In the latter case, dog relocation projects may also contribute to increase pathogen circulation in previously non-endemic regions, and eventually their transmission, when a proper vector is present (Otranto et al. 2017; Mendoza-Roldan et al. 2020).

Dogs also contribute to the circulation of certain tick species, such as *Rhipicephalus sanguineus* sensu lato (s.l.), as well as other more generalist tick species (e.g., *Ixodes ricinus*) which are well recognized as vectors of pathogens to animals and humans (Otranto et al. 2009a).

Therefore, canine vector-borne diseases (CVBDs) caused by a wide range of pathogens, including viruses, bacteria, protozoa, and helminths, are of veterinary importance and may represent a public health issue (Otranto et al. 2009a, 2017). Data on the occurrence of CVBDs are reported in a few countries of the Middle East such as Iraq (Otranto et al. 2019), Turkey (Kirkan et al. 2013), and Qatar (Alho et al. 2017), making their impact on animal and human populations difficult to estimate, which ultimately impairs the implementation of preventive strategies for minimizing the risk of infections. For example, the occurrence of VBPs has been documented only in a few regions and on limited number of dogs in Iran and Pakistan (Khazeni et al. 2013; Khedri et al. 2014; Motaghipisheh et al. 2016; Ahmad et al. 2018a; Mohebali et al. 2018; Barati and Razmi 2018; Azari-Hamidian et al. 2019). In this perspective, the aim of this study was to investigate the prevalence of VBDs in sheltered and owned dogs from five Iranian provinces and from Punjab in Pakistan in order to fill gaps in the knowledge of VBPs in these areas.

## Materials and methods

### Sample collection

**Fig. 1:**
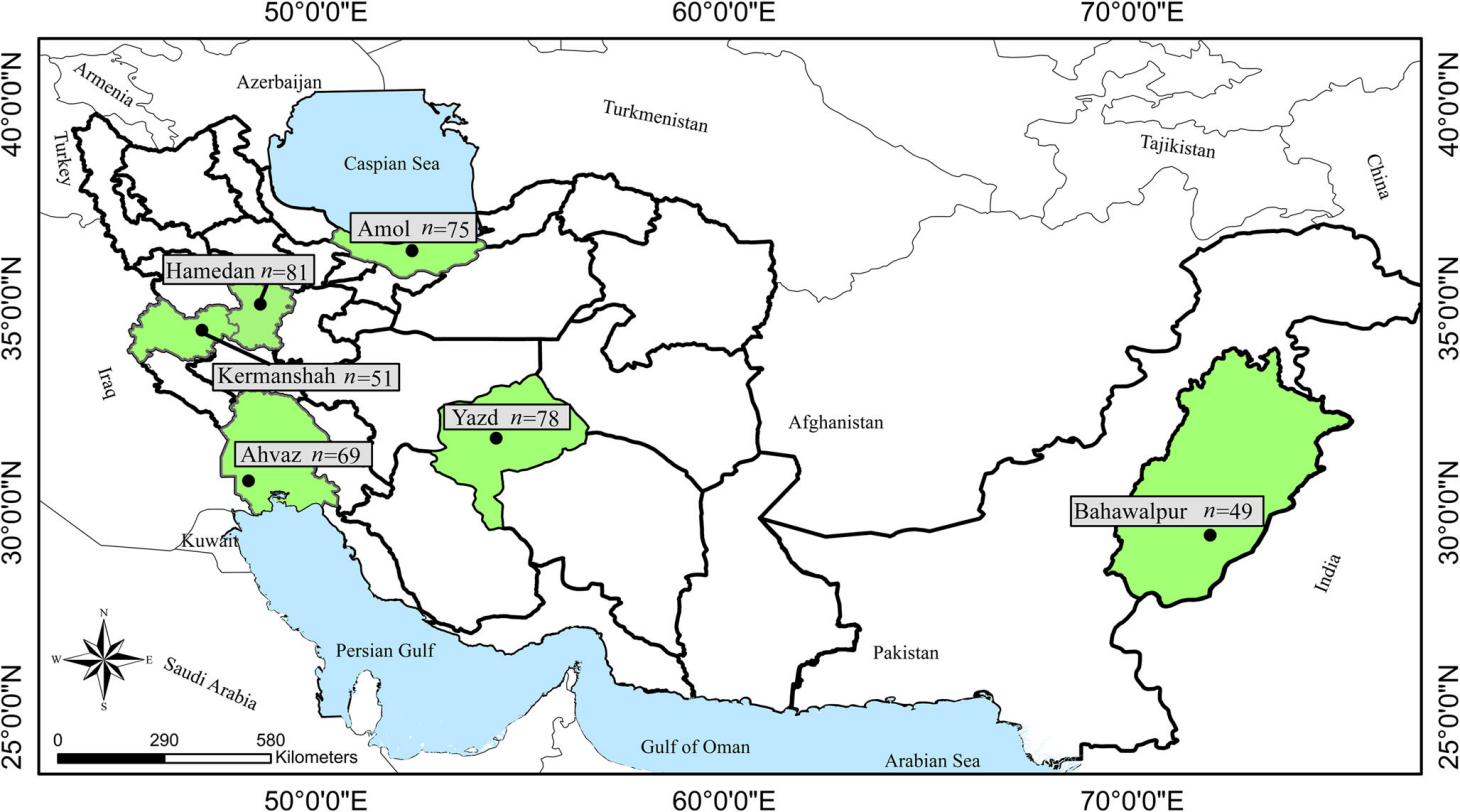
Samples were collected from different provinces in Iran and Pakistan

From October 2018 to November 2019, blood samples (1.5–5 ml) were collected from cephalic or saphenous vein of 403 dogs (i.e., 357 sheltered and 46 privately owned dogs) from 5 cities in 5 provinces of Iran including Amol in the North (*n* = 75), Hamedan (*n =* 81) and Kermanshah (*n =* 51) in the west, Yazd in the center (*n =* 78), Ahvaz in the south-west (*n =* 69), and from Bahawalpur in the eastern province of Punjab, Pakistan (*n =* 49) (Fig. 1). Animal data (i.e., age, sex, breed, neutering status, and province) were recorded. All dogs stayed outside overnight. Dogs were grouped according to age in ≤1 year (G1), >1 to  <5 years (G2), and ≥ 5 years (G3). The blood of dogs was collected with permission of the Ethical Committee of Hamedan University of Medical Sciences, Iran (code: IR.UMSHA.REC.1398.124).

### Serological testing

Serum samples from 354 out of 403 dogs were tested for anti-*L. infantum* antibodies by using an immunofluorescence antibody test (IFAT), as described elsewhere (Otranto et al. 2009c). Promastigotes of *L. infantum* zymodeme MON-1 were used as antigen. Samples were scored as positive when they produced a clear cytoplasmic and membrane fluorescence of promastigotes at a cut-off dilution of 1:80. Positive sera were titrated by serial dilutions until negative results were obtained.

### DNA extraction, PCR protocols, and sequencing

Genomic DNA was extracted from 200 μl aliquots of EDTA-treated blood samples using GenUP DNA Kit (Biotechrabbit, Berlin, Germany) following the manufacturer’s instructions. All samples were tested for the presence of *Anaplasma* spp., *Ehrlichia* spp., *Rickettsia* spp., *Babesia* spp*.*, *Hepatozoon* spp., and filarioid DNA by using conventional PCR (cPCR). In particular, for the detection of *Rickettsia* spp., DNA samples were firstly tested by PCR targeting a partial sequence of the gene citrate synthase (*gltA*) and positive samples were further tested by a second PCR targeting a fragment of the outer membrane protein A (*ompA*) gene. All primers and cPCR protocols used for the detection of VBPs are summarized in Table 1. *Leishmania* spp. and *Coxiella burnetii* were detected by using real-time PCR (qPCR) and positive samples were further tested by cPCR. For all reactions, DNA of pathogen-positive blood samples served as a positive control. Amplified PCR products were visualized in 2% agarose gel stained with GelRed (VWR International PBI, Milan, Italy) by GelLogic 100 gel documentation system (Kodak, NY, USA). The cPCR amplicons were sequenced using the Big Dye Terminator v.3.1 chemistry in a 3130 Genetic Analyzer (Applied Biosystems, CA, USA). Nucleotide sequences were edited, aligned, and analyzed using the Geneious platform version 7.0 (Biomatters Ltd., Auckland, New Zealand) (Kearse et al. 2012) and compared with those available in the GenBank® database using the Basic Local Alignment Search Tool (http://blast.ncbi.nlm.nih.gov/Blast.cgi).

**Table 1:** Primers, target genes, and PCR conditions used in this study

Pathogen	Primers	Target gene	Product size (bp)	Reference
*Ehrlichia* spp*./Anaplasma* spp*.*	EHR16SD: GGTACCYACAGAAGAAGTCC EHR16SR: TAGCACTCATCGTTTACA GC	16S rRNA	345	Martin et al. (2005)
*Babesia* spp./*Hepatozoon* spp.	Piroplasmid-F: CCAGCAGCCGCGGTAAATTC Piroplasmid-R: CTTTCGCAGTAGTTYGTCTTTAACAAATCT	18S rRNA	350–400	Tabar et al. (2008)
*Rickettsia* spp.	CS-78F: GCAAGTATCGGTGAGGATGTAAT CS-323R: GCTTCCTTAAAATTCAATAAATCAGGAT	*gltA*	401	Labruna et al. (2004)
	Rr190.70F: ATGGCGAATATTTCTCCAAAA Rr190.701R: GTTCCGTTAATGGCAGCATCT	*opmA*	632	Regnery et al. (1991)
*Coxiella burnetii*	Cox-F: GTCTTAAGGTGGGCTGCGTG	*IS1111*	295	Klee et al. (2006)
	Cox-R: CCCCGAATCTCATTGATCAGC			
	Cox-TM: FAM-AGCGAACCATTGGTATCGGACGTT-TAMRA-TATGG			
*Leishmania* spp.	LGITSF2: GCATGCCATATTCTCAGTGTC LGITSR2: GGCCAACGCGAAGTTGAATTC	ITS-2	383-450	de Almeida et al. (2011)
	LEISH-1: AACTTTTCTGGTCCTCCGGGTAG LEISH-2: ACCCCCAGTTTCCCGCC Probe: FAM-AAAAATGGGTGCAGAAAT	kDNA minicircle	120	Francino et al. (2006)
Nematodes	NTF: TGATTGGTGGTTTTGGTAA NTR: ATAAGTACGAGTATCAATATC	*cox*1	648	Casiraghi et al. (2001)

### Phylogenetic analysis

For phylogenetic analyses, the 18S rRNA and *gltA* representative sequence types (STs) of *Hepatozoon* spp. and *Rickettsia* spp., respectively, and the corresponding sequences available from the GenBank® database were included. Phylogenetic relationships were inferred using the maximum likelihood (ML) method based on Hasegawa-Kishino-Yano model (Hasegawa et al. 1985) for *Hepatozoon canis* analysis and Tamura 3-parameter model (Tamura 1992) for *Rickettsia* spp. Gamma distribution (+G) was used to model evolutionary rate differences among sites selected by best-fit model (Nei and Kumar 2000). Evolutionary analyses were conducted on 8000 bootstrap replications using the MEGA X software (Kumar et al. 2018). Homologous sequences from *Adelina bambarooniae* (accession nos. AF494058) as well as *Rickettsia typhi* and *Rickettsia prowazekii* (accession nos. U59714 and U59715) were used as outgroups for *Hepatozoon* spp. and *Rickettsia* spp. phylogenetic trees, respectively.

### Statistical analysis

Exact binomial 95% confidence intervals (CIs) were established for proportions. The chi-square and Fisher tests were used to compare proportions with a probability *p* value < 0.05 regarded as statistically significant. Analyses were done using the GraphPad Prism version 8.0.0 (GraphPad Software, San Diego, CA, USA).

## Results

Out of 403 dogs tested, 189 (46.9%; 95% CI 42.1–51.9) scored positive to at least one pathogen. Data on sex, age, and location of animals are reported in Table 2 along with number and percentage of dogs positive to *H. canis*, the most frequently detected pathogen species (41.4%; 95% CI 36.7–46.3) and seropositive to antibodies against *L. infantum* (9.6%; 95% CI 6.9–13.1). The prevalence of infection by other pathogens including *Anaplasma platys*, *Ehrlichia canis*, spotted-fever group (SFG) *Rickettsia* spp., *Babesia* spp., and *L. infantum* is reported in Table 3. No DNA of filarioids or *C. burnetii* was amplified. Co-infections with *H. canis* and *A. platys* were the most frequent (4.9%, *n =* 20), followed by *H. canis* and *E. canis* (1.4%, *n =* 3); *H. canis* and *Rickettsia* spp. (1.4%, *n =* 3); *H. canis* and *L. infantum* (0.3%, *n =* 1); and *H. canis*, *E. canis*, and *Rickettsia* spp. (0.3%, *n =* 1). The *ompA* gene was not amplified in any of the samples positive to *Rickettsia gltA* gene. Out of 354 serum samples, 34 (9.6%) were positive to anti-*L. infantum* antibodies with titers of 1:80 (*n* = 14), 1:160 (*n* = 15), 1:320 (*n =* 3), 1:640 (*n =* 1), and 1:1280 (*n =* 1).

**Table 2:** Number and percentage of dogs positive to *Hepatozoon canis* DNA (*n* = 403) and antibodies against *Leishmania infantum* (*n* = 354) according to their sex, age, keeping condition, and sampling area. (*NA* indicates data not available)

Variables		*Hepatozoon canis*	*Leishmania infantum*
		Pos/total (%; 95% CI)	Pos/total (%; 95% CI)
Sex			
Male		73/161 (45.3; 37.6–53.1)	12/122 (9.8; 5.6–16.7)
Female		92/240 (38.3; 32.3–44.8)	22/230 (9.6; 6.2–14.1)
No data		2/2 (100)	0/2 (0)
Age			
≤ 1 year		34/84 (40.5; 30.3–51.2)	4/59 (6.8; 2.3–16.7)
> 1 year to < 5 years		96/241 (39.8; 33.8–46.3)	18/219 (8.2; 5.2–12.7)
> 5 years		34/74 (45.9; 34.6–57.5)	12/74 (16.2; 9.3–26.3)
No data		3/4 (75.0; 24.8–98.2)	0/2 (0)
Keeping condition			
Privately owned		2/46 (4.3; 0.8–14.9)	8/46 (17.4; 9.0–30.7)
Shelter		165/357 (46.2; 41–51.5)	26/308 (8.4; 5.7–12.1)
Geographical origin (country/city)			
Iran	Ahvaz	34/69 (49.3; 37.6–60.9)	6/69 (8.7; 3.8–17.9)
	Amol	32/75 (42.7; 31.9–54.1)	19/75 (25.3; 16.5–36.6)
	Kermanshah	40/51 (78.4; 64.8–87.9)	0/51 (0)
	Hamedan	15/81 (18.5; 11.2–28.3)	9/81 (11.1; 5.7–20.2)
	Yazd	15/78 (19.2; 11.7–29.4)	0/78 (0)
Pakistan	Bahawalpur	31/49 (63.3; 48.9–75.7)	NA
Total		167/403 (41.4; 36.7–46.4)	34/354 (9.6; 6.9–13.1)

**Table 3:** Number (percentage) of dogs molecularly positive to vector-borne pathogens according to their location

Country	Province	Total number of dogs infected with each pathogen (%)				
		*Anaplasma platys*	*Ehrlichia canis*	*Rickettsia* spp.	*Babesia vogeli*	*Leishmania infantum*
Iran	Ahvaz (69)	1 (1.4)	1 (1.4)	8 (13)^		
	Amol (75)	2 (2.7)	1 (1.3)			
	Hamedan (81)	-			4 (4.9)	
	Kermanshah (51)	10 (19.6)				1 (2)
	Yazd (78)	-	-			
Pakistan	Bahawalpur (49)	13 (26.5)	12 (24.5)	1 (2)°		
Total (403)		26 (6.4)	14 (3.4)	9 (2.2)	4 (0.99)	1(0.25)

^*n* = 4, *Rickettsia monacensis*; *n* = 2, *Rickettsia helvetica*; *n* = 2, *Rickettsia heilongjiangensis/Rickettsia raoultii/Rickettsia slovaca*

°*n* = 1, *Rickettsia conorii*/*Rickettsia honei*/*Rickettsia raoultii*

The risk of *H. canis* infection in dogs was significantly associated with their keeping conditions and the geographical areas, whereas the risk of *L. infantum* exposure only with the geographical areas. In particular, sheltered dogs were related to higher risk of *H. canis* infection than owned dogs (*χ*^2^ = 29.4, df = 1, *p* < 0.00001). Based on location, the highest prevalence of *H. canis* infection (i.e., 78.4%) was recorded in Kermanshah in Iran followed by Bahawalpur in Pakistan (63.3%) and both percentages were significantly higher than those recorded in other cities (*p* < 0.05). Dogs from Amol in Iran were more exposed to *L. infantum* than those in the other Iranian provinces (*p* < 0.05) (Table 2). No statistical association was found between *H. canis* infection or *L. infantum* exposure in dogs and their sex and age. The region with the highest pathogen diversity was Ahvaz in Iran where a high prevalence of *Rickettsia* spp. infection was recorded (13%) (Table 3).

Consensus sequences for each detected pathogen displayed 99–100% nucleotide identity with those available in the GenBank® database. In particular, *Babesia vogeli* (*n =* 4) nucleotide similarity was 99.3–99.6% with MT499357. In addition, five STs were identified for *H. canis* (ST1, *n =* 6, 100% identity with MN393911; ST2, *n =* 10, 99.7% with MK673827; ST3, *n =* 24, 100% with KX880505; ST4, *n =* 39, 100% with MT499354; ST5, *n =* 88, 100% with MK673850) and *L. infantum* 100% identity was recorded with MK496879. *Anaplasma platys* sequences (*n =* 26) and *E. canis* (*n =* 14) had 100% nucleotide identity with MN922611 and MN922610, respectively. As far as *Rickettsia* spp., four samples had 100% nucleotide identity with *Rickettsia monacensis* (KC993860) and two had 99.7% identity with *Rickettsia helvetica* (U59723). One sample from a dog in Pakistan was 100% identical to *Rickettsia conorii* (U59730)/*Rickettsia honei* (U59726)/*Rickettsia raoultii* (CP019435), and two other samples from dogs in Ahvaz (Iran) were 100% identical to *Rickettsia heilongjiangensis* (CP002912)/*Rickettsia raoultii* (DQ365803)/*Rickettsia slovaca* (U59725).

**Fig. 2:**
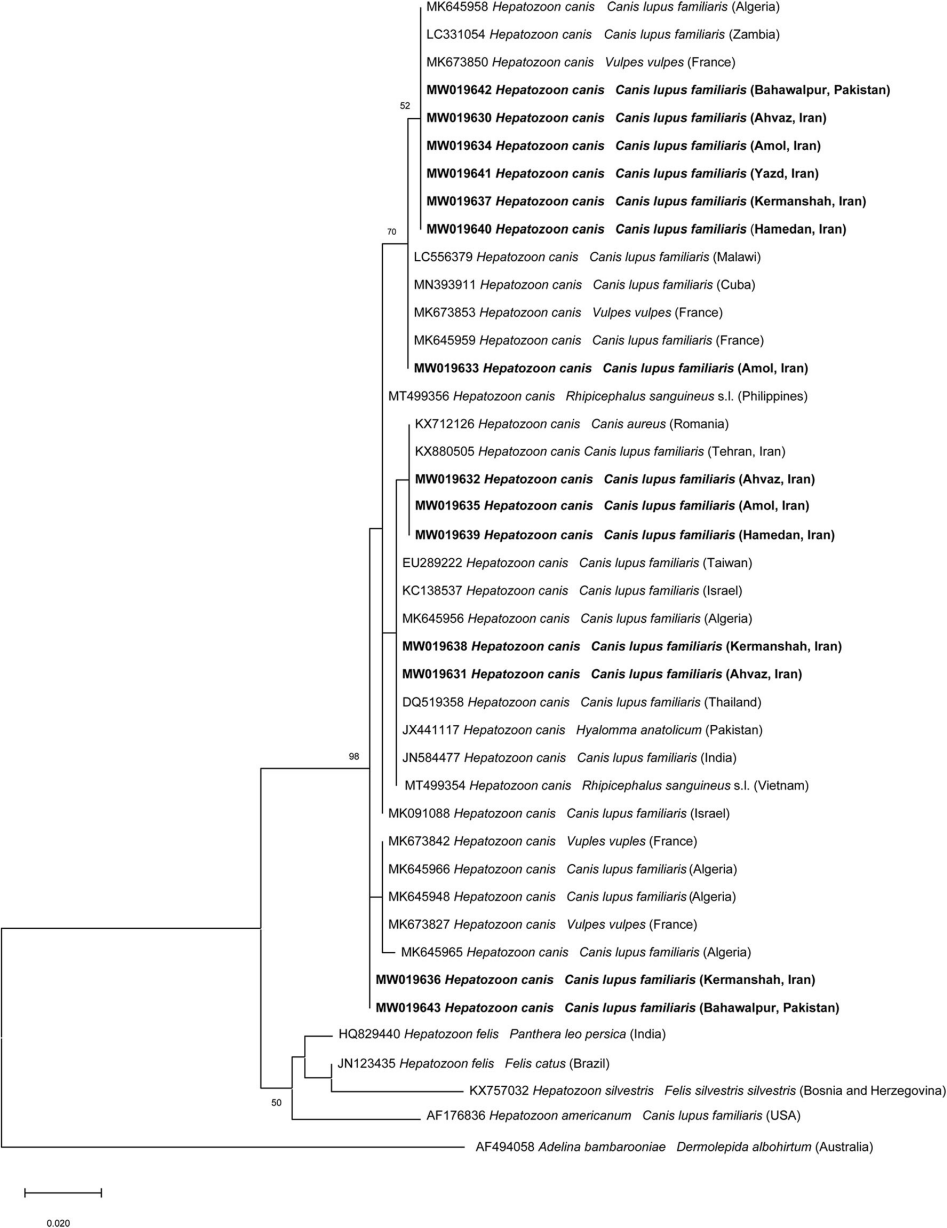
Phylogenetic relationship of *Hepatozoon* spp. sequences isolated in this study (in bold) to other *Hepatozoon* spp. based on a partial sequence (327 bp) of the 18S rRNA gene. The analyses were performed using a maximum likelihood with Hasegawa-Kishino-Yano model. A gamma distribution was used to model evolutionary rate differences among sites. Homologous sequence from *Adelina bambarooniae* (accession nos. AF494058) was used as the outgroup

**Fig. 3:**
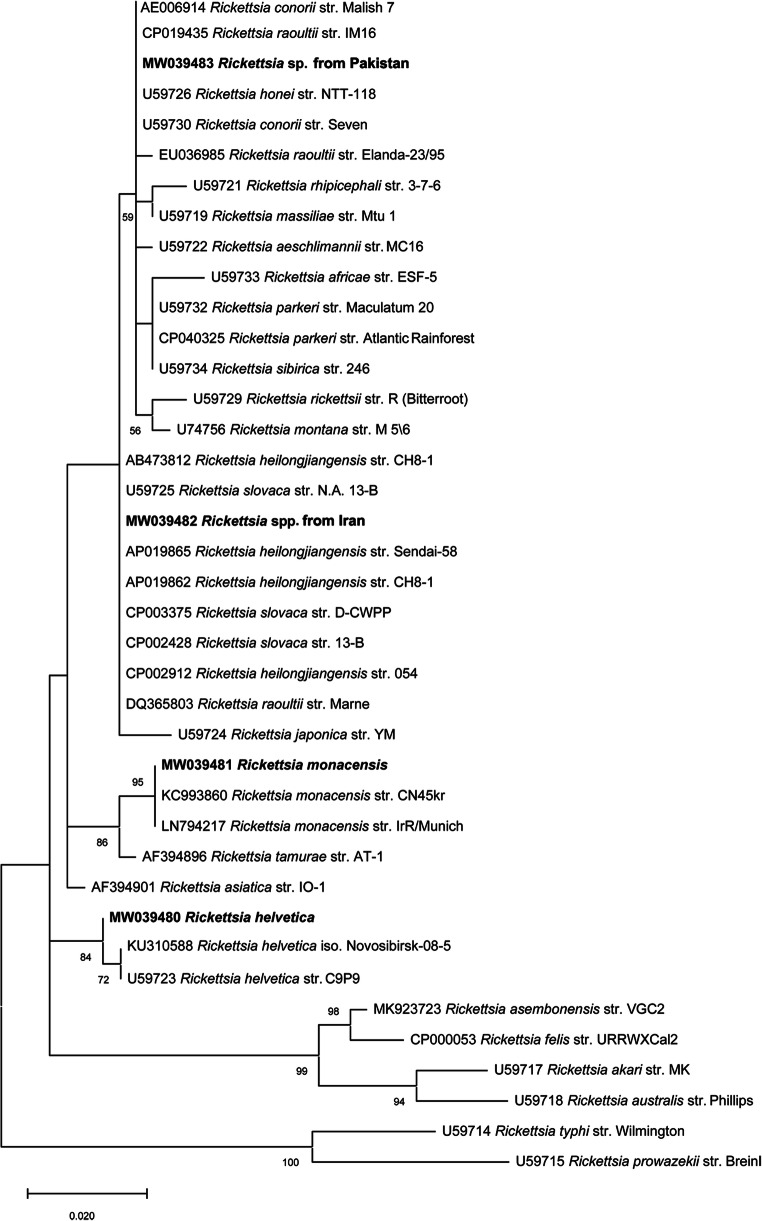
Phylogenetic relationship of *Rickettsia* spp. sequences isolated in this study (in bold) to other *Rickettsia* strains based on a partial sequence (345 bp) of the *gltA* gene**.** The analyses were performed using a maximum likelihood method with Tamura 3-parameter model. A gamma distribution was used to model evolutionary rate differences among sites. Homologous sequences from *Rickettsia typhi* (accession nos. U59714) and *Rickettsia prowazekii* (accession nos. U59715) were used as the outgroups

Molecular identification of nucleotide sequences for *Rickettsia* spp. and *H. canis* was supported by the distinct separation of species-specific clades inferred from the phylogenetic analyses. The ML tree of *H. canis* grouped all representative STs in a large clade supported by high bootstrap value (i.e., 98%), to the exclusion of other species of *Hepatozoon* (Fig. 2). The ML tree based on the partial *gltA* gene sequences of *Rickettsia* spp. showed that all sequences, herein detected, clustered in well-supported clades with other SFG rickettsiae (Fig. 3). Representative sequences of pathogens detected in this study were deposited in the GenBank® database under the accession numbers MW019628 and MW019629 for *B. vogeli*, MW019630-MW019643 for *H. canis*, MW019670 and MW019671 for *A. platys*, MW019672 and MW019673 for *E. canis*, MW039480 for *R. helvetica*, MW039481 for *R. monacensis*, MW039482 and MW039483 for *Rickettsia* spp., and MW074300 for *L. infantum*.

## Discussion

The high frequency of CVBD-causing pathogens reported herein indicates that dog populations from Iran and Pakistan are exposed to multiple pathogens, including some of zoonotic importance, thus posing a risk not only to dogs, but also to public health. Many of the herein detected pathogens (i.e., *A. platys*, *E. canis*, *B. vogeli*, and *H. canis*) are transmitted by *Rh. sanguineus* s.l. (Latrofa et al. 2014), the most common tick species found on dogs in Iran and Pakistan (Mosallanejad et al. 2012; Cabezas-Cruz et al. 2019). The high prevalence of *H. canis* in all the provinces of Iran as well as from Bahawalpur in Pakistan suggests that *Rh. sanguineus* s.l. is highly prevalent in these areas. After the first detection of *H. canis* gametocytes in a blood smear of an Iranian dog (Khoshnegah et al. 2009), the prevalence of this infection was reported only in dogs from northern half of Iran ranging from 1.6 to 23% (Amoli et al. 2012; Dalimi et al. 2017; Soltani and Dalimi 2018; Barati and Razmi 2018). Conversely, the prevalence herein recorded for *H. canis* in Pakistan (i.e., 41.4%) was similar to that of farm dogs recorded in Punjab districts (45.5%, Ahmad et al. 2018a). Scientific data indicates that under certain circumstances, such as in poor socioeconomic settings, the high environmental infestation of arthropod vectors and the absence of preventive strategies in dogs and in the environment concur to increase the incidence of CVBD (Otranto et al. 2017). Indeed, similar high prevalence of *H. canis* infections has been reported in dogs (37.8%) and in *Rh. sanguineus* s.l. ticks (42.5%) from India (Manoj et al. 2020) as well as in dogs from Iraq (33%) (Otranto et al. 2019). Although *Rh. sanguineus* s.l., *Rhipicephalus bursa* and *Rhipicephalus annulatus* are the common species infesting dogs in the studied area (Jamshidi et al. 2012; Akhtardanesh et al. 2016; Ali et al. 2019; Cabezas-Cruz et al. 2019), the circulation of other competent tick vectors cannot be ruled out (Murata et al. 1995; Rubini et al. 2009; Najm et al. 2014; Giannelli et al. 2017). In addition, the significantly higher occurrence of dogs infected by *H. canis* in Kermanshah in Iran (78.4%) and Bahawalpur in Pakistan (63.3%) could be related to the high population density of tick vectors in these shelters observed during the dog sampling. The risk for sheltered dogs to be more infected by *H. canis* than owned dogs suggests that in this environment, the animals are more exposed to the vectors. Indeed, preventive measures such as ectoparasite repellents are not commonly employed in sheltered dogs in Iran and Pakistan because of the limitation in budget of non-governmental organization which mainly aim to feed and protect dogs from culling program (personal observations of AS and MMA). Conversely, *B. vogeli* was found in only few dog samples (i.e., 1%) as recorded in other studies from Iran (Alborzi et al. 2013; Akhtardanesh et al. 2016; Bigdeli and Namavari 2017) and Pakistan (Ahmad et al. 2018b). Also, *Babesia gibsoni* was reported causing canine babesiosis in these two countries (Akhtardanesh et al. 2016; Ahmad et al. 2018b).

In Iran, the first study on leishmaniosis in carnivores was conducted in 1982 with a seroprevalence of 2.4% in jackals and 3% in dogs from the northern part (Hamidi et al. 1982). Afterwards, *L. infantum* infection was reported in domestic and wild animals in most of the regions of Iran with differences in frequency probably related to geographic regions, environmental conditions, and dog population (Shokri et al. 2017; Mohebali et al. 2018). The seropositivity to *L. infantum* in dogs from Iran herein detected (9.6%) confirms the circulation of infected sand fly vector (Yaghoobi-Ershadi 2016), thus the risk of dogs as well as humans to be exposed to their bites and to be infected. Differences in climatic factors may affect the sand fly population (Cheghabalaki et al. 2019) in terms of species composition and abundance, thus leading to a higher risk of infection in dogs living in some provinces. Indeed, the highest seroprevalence (25.3%) was recorded in dogs from Amol, located in the north of Iran with a Mediterranean climate vs no infected dog in Yazd and Kermanshah which are regions characterized by hot and dry climate. The detection of *L. infantum* DNA only in one dog may also be related to the use of the blood, which is a convenient type of sample but poor as a parasite source (Maia et al. 2009; Otranto et al. 2009c).

Canine monocytic ehrlichiosis by *E. canis* and canine cyclic thrombocytopenia by *A. platys* affect dogs worldwide, varying from asymptomatic infections or with mild symptoms to a severe illness (Otranto et al. 2009b). Although the prevalence of these infections is scantly reported in the examined areas, the finding of *E. canis* in dogs from Iran and Pakistan provinces is not surprising since this bacterium is transmitted by *Rh. sanguineus* s.l. A few seroprevalence studies (Akhtardanesh et al. 2010) as well as molecular detection of *E. canis* (Motaghipisheh et al. 2016; Malik et al. 2018) in blood samples confirm that dogs from the examined areas are exposed to the pathogen. Conversely, the first report of *A. platys* in both countries with higher prevalence than *E. canis* (6.4% vs 3.4%) indicates that this pathogen may affect dogs living mainly in rural area and in shelters. While the unsuccessful amplification of the *ompA* gene may represent a limitation of the study, some sequences obtained from dogs from Iran had high (> 99%) sequence identity with *R. helvetica* and *R. monacensis*. Although the pathogenicity of *R. helvetica* and *R. monacensis* in dogs is unknown, both species may cause severe diseases in humans as reported in Europe and Southeast Asia for *R. helvetica* (Nilsson et al. 1999; Fournier et al. 2004; Nilsson et al. 2010) and in Spain and Italy for *R. monacensis* (Jado et al. 2007; Madeddu et al. 2012). Data on SFG rickettsiae in dogs and their ectoparasites in the Middle East are scant (Baneth et al. 1998; Harrus et al. 2011; Levin et al. 2011; Kirkan et al. 2013; Parola et al. 2013; Orkun et al. 2014). Our findings suggest the occurrence of SFG rickettsiae in dogs from Iran and Pakistan, but further studies are needed to confirm the identity of these microorganisms and also to understand the potential risks for public health.

In this study, none of the tested dogs scored positive to *C. burnetii* although this bacteria was serologically diagnosed in a domestic dog in Ahvaz, Iran (Rezaei et al. 2016). Indeed, Q fever is endemic in Iran and Pakistan with high prevalence among human patients and domestic ruminants (Ullah et al. 2019; Esmaeili et al. 2019; Mohabbati Mobarez et al. 2017). In fact, it is acknowledged that ruminants (cattle, sheep, and goats) are the most important reservoirs of *C. burnetii* for human infection (Angelakis and Raoult 2010).

In conclusion, the herein reported data provide more knowledge of CVBDs in these countries suggesting that different pathogens as well as arthropod vectors circulate among animal populations. Of the detected pathogens, SFG rickettsiae are of great relevance for their pathogenicity to humans. Considering the occurrence of infection by zoonotic pathogens in dogs and their close relationship with humans, effective control strategies are advocated for minimizing the risk of infection in animals as well as in humans.
